# Long Island Veterinary Society

**Published:** 1890-12

**Authors:** D. S. Breslin

**Affiliations:** Secretary


*Long Island Veterinary Society.*—A regular meeting of the Long Island Veterinary Society was held on November 19th, 1890, at No. 74 Adams Street, the President, Dr. George H. Berns, in the chair. The minutes of the previous meeting were read and approved. The following members were present: Drs. George H. Berns, George F. Bowers, William H. Pendry, Roscoe R. Bell, Samuel Atchinson and D. S. Breslin. The Board of Censors made no report.

The next order of business was reading of papers. Dr. George H. Berns presented a paper on Osteo-Perosis, see page 714.

The reading of the paper was followed by an interesting discussion by the members, after which a vote of thanks was tendered to the essayist.

The next order of business was the nomination of officers to be elected at the December meeting. The following gentlemen were placed in nomination: For president, Dr. R. R. Bell and Dr. William H. Pendry; for vice-president, Dr. Samuel Atchison; for treasurer, Dr. George F. Bowers; for secretary, Dr. D. S. Breslin; Board of Censors: Dr. George H. Berns, Dr. Philip Newman; Dr. H. Hausman, Dr. Thomas M. Buckley: Dr. J. F. Mustoe.

The Chair appointed Dr. William H. Pendry as essayist for December meeting. The meeting then adjourned.

D. S. BRESLIN, D.V.S., *Secretary.*

